# Patient‐centered design of secure messaging for dementia risk in primary care

**DOI:** 10.1002/alz70858_107569

**Published:** 2025-12-26

**Authors:** Diana Summanwar, Deanna R Willis, Nicole R Fowler

**Affiliations:** ^1^ Indiana University School of Medicine, Indianapolis, IN, USA; ^2^ Indiana University Center for Aging Research, Indianapolis, IN, USA

## Abstract

**Background:**

Primary Care Providers (PCPs) are often the first point of contact for older adults with mild cognitive impairment (MCI) and Alzheimer's disease and related dementias (ADRD). However, resource and time constraints hinder the ability of primary care practices to perform early detection and timely intervention. To address this gap, scalable models are needed to identify individuals at risk for MCI and ADRD, notify them of their elevated risk, and engage them in care pathways for evaluation, treatment, and research participation.

**Method:**

Using a user‐center design approach, we engaged primary care patients in co‐developing a messaging strategy aimed at effectively reaching individuals at elevated risk for cognitive impairment, assessing whether they experience any subjective cognitive concerns, and gauging their interest in connecting to a clinical Brain Health Navigator for further evaluation and support.

**Result:**

We enrolled a diverse cohort of 16 primary care patients in a user‐centered design session, during which key themes emerged as essential willingness to engage with the outreach messaging. Patients emphasized the importance of recognizable connection to their PCP, such as including their PCP's name in the initial message. They also preferred timely outreach, ideally aligned with upcoming primary care appointments, to enhance relevance and engagement. In addition, clarity about why they were being contacted regarding brain health and how a brain health navigator could support their care journey was critical to fostering trust and willingness to pursue next steps. Based on these insights, draft messages were developed.

**Conclusion:**

Applying user‐centered design techniques to brain health messaging helps identify key patient engagement factors necessary for the development of scalable, technology‐driven outreach strategies. This approach enhances early detection efforts in primary care patients and supports timely evaluation and intervention.

